# Acute disseminated encephalomyelitis following coronavirus disease vaccination

**DOI:** 10.1590/0037-8682-0166-2022

**Published:** 2022-08-05

**Authors:** Ronaldo Gonçalves Pereira, Rafael Teixeira de Hollanda Lima, Bruno Niemeyer de Freitas Ribeiro

**Affiliations:** 1 3D Diagnóstico / Hospital Rede Casa, Departamento de Radiologia, Rio de Janeiro, RJ, Brasil.; 2 Labs A+/Grupo Fleury, Rio de Janeiro, RJ, Brasil.; 3 Instituto Estadual do Cérebro Paulo Niemeyer, Departamento de Radiologia, Rio de Janeiro, RJ, Brasil.

**FIGURE 1:**
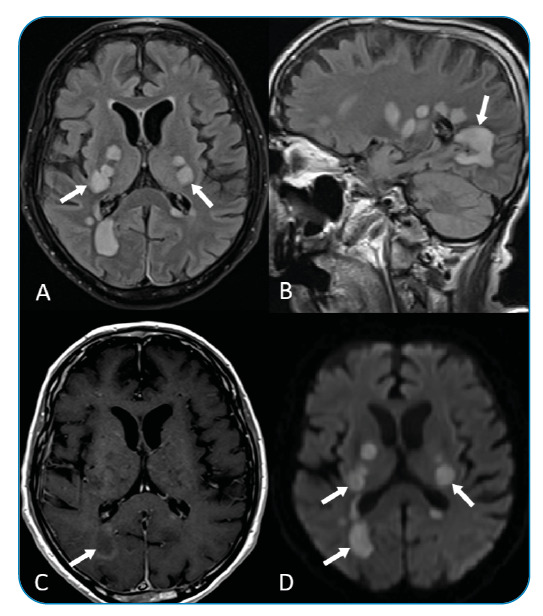
Fluid-attenuated inversion recovery magnetic resonance imaging (MRI) findings in the **(A)** axial and **(B)** sagittal views showing multiple hyperintense white matter lesions and basal nuclei bilaterally (arrows). **(C)** Contrast-enhanced T1-weighted MRI findings showing an incomplete arc of contrast enhancement (arrows). **(D)** MRI diffusion-weighted imaging showing hyperintense lesions (arrows).

A 76-year-old man presented to our hospital with bilateral progressive amaurosis and consciousness disorder 4 weeks after he had received the Sinovac coronavirus disease (COVID-19) vaccine. He had no notable medical history, mildly elevated levels of cerebrospinal fluid protein, and a normal blood cell count. Brain magnetic resonance imaging findings revealed multiple white-matter lesions bilaterally (Figure 1A-D). The suspected diagnosis was acute disseminated encephalomyelitis. This disease usually develops following viral infection or vaccination and is characterized by monophasic acute inflammation and demyelination of the white matter, typically 1-2 weeks after exposure. Few reports have described the occurrence of acute disseminated encephalomyelitis following COVID-19 vaccination^1^ ^,^ ^2^.
